# An evidence-base for the implementation of hospital-based palliative care programs in routine cancer practice: A systematic review

**DOI:** 10.1177/02692163231186177

**Published:** 2023-07-08

**Authors:** Farwa Rizvi, Helen Elizabeth Wilding, Nicole M Rankin, Roslyn Le Gautier, Lorna Gurren, Vijaya Sundararajan, Kylee Bellingham, Joyce Chua, Gregory B Crawford, Anna K Nowak, Brian Le, Geoff Mitchell, Sue-Anne McLachlan, Tanara Vieira Sousa, Robyn Hudson, Maarten IJzerman, Anna Collins, Jennifer Philip

**Affiliations:** 1 Palliative Medicine, University of Melbourne, Parkville, Victoria, Australia; 2 Library Service, St Vincent’s Hospital Melbourne, Fitzroy, Victoria, Australia; 3 Evaluation and Implementation Science Unit, Centre for Health Policy, Melbourne School of Population and Global Health, University of Melbourne, Parkville, Victoria, Australia; 4 Deakin University, Melbourne, Victoria, Australia; 5 Dublin City University, Dublin, Ireland; 6 La Trobe University, Melbourne, Victoria, Australia; 7 Department of Medicine, St Vincent’s Hospital, Melbourne Medical School, Fitzroy, Victoria, Australia; 8 Research Nurse Palliative Medicine, St Vincent’s Hospital Melbourne, Fitzroy, Victoria, Australia; 9 Peter MacCallum Cancer Centre, Melbourne, Victoria, Australia; 10 Adelaide Medical School, University of Adelaide, Adelaide, South Australia, Australia; 11 Northern Adelaide Local Health Network, Adelaide, South Australia, Australia; 12 Medical School, University of Western Australia, Perth, Western Australia, Australia; 13 Deparment of Palliative Care, Peter MacCallum Cancer Centre, Melbourne, Victoria, Australia; 14 Deparment of Palliative Care, Palliative Care, The Royal Melbourne Hospital, Parkville, Victoria, Australia; 15 University of Melbourne, Parkville, Victoria, Australia; 16 General Practice Clinical Unit, University of Queensland, Brisbane, Queensland, Australia; 17 Oncology and Cancer Services, St Vincent’s Hospital, University of Melbourne, Parkville, Victoria, Australia; 18 Safer Care Victoria, Melbourne, Victoria, Australia; 19 Cancer Health Services Research, University of Melbourne, Parkville, Victoria, Australia; 20 Department of Medicine, St Vincent’s Hospital, University of Melbourne, Parkville, Victoria, Australia; 21 Palliative Medicine, Department of Medicine, St Vincent’s Hospital Melbourne, Victoria, Australia

**Keywords:** Palliative care, quality of life, evidence gaps, cancer care facilities, systematic review, implementation science

## Abstract

**Background:**

Despite global support, there remain gaps in the integration of early palliative care into cancer care. The methods of implementation whereby evidence of benefits of palliative care is translated into practice deserve attention.

**Aim:**

To identify implementation frameworks utilised in integrated palliative care in hospital-based oncology services and to describe the associated enablers and barriers to service integration.

**Design:**

Systematic review with a narrative synthesis including qualitative, mixed methods, pre-post and quasi experimental designs following the guidance by the Centre for Reviews and Dissemination (PROSPERO registration CRD42021252092).

**Data sources:**

Six databases searched in 2021: EMBASE, EMCARE, APA PsycINFO, CINAHL, Cochrane Library and Ovid MEDLINE searched in 2023. Included were qualitative or quantitative studies, in English language, involving adults >18 years, and implementing hospital-based palliative care into cancer care. Critical appraisal tools were used to assess the quality and rigour.

**Results:**

Seven of the 16 studies explicitly cited the use of frameworks including those based on RE-AIM, Medical Research Council evaluation of complex interventions and WHO constructs of health service evaluation. Enablers included an existing supportive culture, clear introduction to the programme across services, adequate funding, human resources and identification of advocates. Barriers included a lack of communication with the patients, caregivers, physicians and palliative care team about programme goals, stigma around the term ‘palliative’, a lack of robust training, or awareness of guidelines and undefined staff roles.

**Conclusions:**

Implementation science frameworks provide a method to underpin programme development and evaluation as palliative care is integrated within the oncology setting.

**What is already known about the topic**?

- The hospital based palliative care services have been ascertained as enabling access to early or timely palliative care. Yet integration of palliative care into existing cancer services remains limited with patients continuing to be referred to palliative care at a late stage, if at all.
- Implementation science offers an approach to underpin the development of integrated palliative care services.

**What this paper adds?**

- Just seven studies utilised implementation frameworks to guide service delivery which included RE-AIM, SELFIE model, Medical Research Council framework, and the quality improvement approach of Define, Measure, Analyze and Improve.
- The main facilitators for palliative care integration included the value of co-design with ‘end users’ of the intervention and the processes of implementation; staff education and training including around processes, communication, and introducing referrals; dedicated communication across the multidisciplinary teams in the hospital. Barriers included lack of time and rapid staff turnover, maintaining fidelity and consistency of the intervention across individual patients and different sites, and a lack of relevant health policy regulations and resources to support palliative care services.

**Implications for practice, theory, or policy**

- A deeper understanding of the enablers and barriers to the success or failure of the previous palliative care implementation efforts and the opportunities provided by a systematic approach as offered by implementation frameworks will benefit the effective establishment and sustainability of future integrated palliative care programs.
- The findings from this systematic review may help stakeholders including policy makers and service providers in allocating precious resources towards planning and systematically implementing integrated palliative care programs in hospital-based cancer services.

## Introduction

Palliative care is increasingly recognised^1^ as a core component of oncology care.^2,3^ Significant evidence from randomised clinical trials has validated the proven benefits of early palliative care for people with advanced cancer including better symptom management,^4^ increased satisfaction with care, and reduced psychological suffering.^5–7^ This has led to a strong global consensus supporting the integration of early palliative care in routine cancer practice.^2,8^

Implementation science is the study of methods and approaches that facilitate the uptake of research into clinical practice.^9^ It includes the identification and response to barriers that block the uptake of proven interventions into care, thereby closing the information gap.^10,11^ There is limited literature to guide the implementation of timely palliative care in cancer care services.^10,12^ and timely referral to hospital based palliative care services remains challenging.^13^ Many clinicians, who oversee the introduction of programs in clinical settings, are unfamiliar with the current implementation science evidence meaning that ‘real-world changes’ do not result.^12^

There is a need for clinical researchers to consider the acceptability, fidelity, and viability of the intervention that is to be implemented, particularly during the initial phases of its development, when its appraisal and utility may be assessed with iterative checks over time.^14^ There is also an opportunity for clinical researchers to take a ‘systems’ approach as available in implementation science to the translation of research findings around early palliative care. This systematic review and narrative synthesis aimed to identify the existing utilisation of implementation science frameworks, to describe the barriers and enablers encountered, and strategies adopted to implement integrated palliative care into hospital-based oncology services.

## Design

A systematic review was conducted with a narrative synthesis approach. Due to the diversity of included study designs (qualitative, mixed methods, pre-post and quasi experimental designs, this systematic review utilised a narrative synthesis approach to presenting the data as guided by Popay et al.^15^ and followed the guidelines by the Centre for Reviews and Dissemination.^16^ This systematic review was registered on the International Prospective Register of Systematic reviews PROSPERO CRD42021252092. Preferred Reporting Items for Systematic Reviews and Meta-Analyses (PRISMA) guidelines were followed for reporting.

### Review aim/questions

The aim of this systematic review was to identify the existing utilisation of implementation science frameworks, and to describe the barriers and enablers encountered and strategies adopted to implement integrated palliative care into hospital-based oncology services.

### Inclusion/exclusion criteria

Inclusion: Studies conducted in English language, on humans 18 years and older, reporting on the process of implementing hospital-based palliative care and oncology integration, including qualitative, quantitative, or mixed methods studies (including studies involving interviews, focus groups, surveys and cross-sectional questionnaires) as well as formal randomised controlled trials or implementation studies published in the year 2010 onwards.^17^ The target clinical group included adult patients with advanced cancer including those with distant metastases, or locally advanced cancer that is life limiting.^2^

Exclusion: Publications classified as book reviews or sections, case reports, comments, conference abstracts, posters or oral presentations, conference reviews, dissertations, editorials, lectures, letters or review papers were excluded.

### Information sources/data sources

Publications were identified through systematic searches of six bibliographic databases, conducted on 12 March 2021: Ovid MEDLINE(R) ALL 1946 to March 10, 2021; Embase 1974 to 2021 March 10 (Ovid); Ovid Emcare 1995 to 2021 Week 08; APA PsycInfo 1806 to March Week 1 2021 (Ovid); CINAHL (EBSCOhost) and Cochrane Library (Wiley). The reference lists of included papers were also searched. An updated additional search was conducted in Medline Ovid from 2021 to 2023 (current) for any pertinent papers published recently. In addition, a hand search was conducted in Google Scholar.

### Search strategy

Search strategies were developed by a medical librarian in consultation with a topic expert. Potential search terms were identified through text mining in PubMed PubReminer^18^ using the query: (‘palliative care’[Mesh:NoExp] OR palliative[tiab]) AND (neoplasms[Majr] OR cancer*[tiab]) AND hospital*[tiab] AND ((implement*[tiab] OR integrat*[tiab]) OR ‘implementation science’[Mesh:NoExp]). Forty publications identified from those results were further analysed using Yale Text Analyser.^19^ Search terms retrieved through text mining were extensively tested for usefulness and relevance in Ovid Medline to develop the final search strategy.

Final search strategies combined the general concepts of Palliative Care AND Cancer AND hospital AND Implementation using a combination of subject headings and text words. Searches were limited to English language publications, but no date limits were applied. An initial search was developed for Ovid Medline (Supplemental File 1) and then adapted for other databases adjusting subject headings and syntax as appropriate (Supplemental Appendix 1). Search syntax used in the Ovid databases was adapted for CINAHL (EBSCOhost) and Cochrane (Wiley) using the Polyglot Search Translator.^20^

### Study selection process

Search results were exported to EndNote bibliographic management software^21^ and duplicates were removed by one author. In accordance with inclusion and exclusion criteria (Table 1), records were screened on publication type by the same author, and book reviews; books and book sections; case reports; comments; conference abstracts; conference reviews; dissertations; lectures and letters were excluded.

**Table 1.** Inclusion/exclusion criteria.

Inclusion criteria	Exclusion criteria
● Qualitative, quantitative studies or mixed methods studies for palliative care as well as formal randomised controlled or implementation studies published in year 2010 onwards^a^ including interviews, focus groups, survey, and cross-sectional questionnaires. ● English language ● Humans 18 years and older ● Studies reporting on the process of implementing hospital-based palliative care interventions that were either patient and/or systems level-focused.	● Languages other than English ● Books, book reviews or sections, case reports, comments, conference abstracts, posters or oral presentations, conference reviews, dissertations, editorials, lectures, letters, review papers

a A decision was made to include only those studies published after 2010 based on the development of the field of integrated palliative care following the publication of a landmark palliative care trial (Temel et al.^17^).

All remaining records were loaded into Covidence^22^ systematic review software for screening on title and abstract by a second author and included records were independently screened a second time on title and abstract by another author within Covidence. These two authors made the decision to include only those studies published after 2010 based on the development of the field of integrated palliative care following the publication of Temel et al.,^17^ a landmark study which heralded integrated palliative care using the model of service delivery. This study was helpful in shaping the discourse in literature and the service development that followed.

The remaining records were exported to EndNote and assessed for eligibility on full text by another two independent reviewers. Disagreements and uncertainties around screening outcomes were resolved by an iterative inter-reviewer discussion between the two authors until a consensus was reached.

The following data were independently collected by two authors from included articles in tabulated formats: author(s), date of publication, participant group, study design. Data were also collected for key findings around descriptions of the implementation process including references to implementation frameworks and models and any barriers and enablers to the implementation. The critical quality appraisal for risk-of-bias was conducted by two authors.

### Strategy for data synthesis

A narrative synthesis approach was used to summarise the data. The defining characteristic of narrative synthesis as described by Popay et al.^15^ is that it adopts a textual approach to the process of synthesis to ‘tell the story’ of the findings from the included studies.^15^ The purpose of the narrative synthesis was to organise findings from the included studies in order to provide an assessment of the strength of the evidence for drawing conclusions about the facilitators and/or barriers to implementation identified in the synthesis. The steps towards data synthesis involved two researchers reading the included papers multiple times to facilitate thematic analysis, ascertaining the main, repeating and/or pertinent findings or themes (based on the review question) and/or concepts emerging from the various studies.^15^ Thematic analysis provided the researchers with a means of organising and summarising the main ideas and conclusions across the diverse research studies. Patterns were identified in the sections including methods, results and discussion by reviewing the full-text papers and potential themes were extracted in a table and discussed among the two researchers in order to ensure the context of themes, which were later refined under categories. Any inter researcher disagreement about the themes was resolved through consensus and a third senior researcher’s advice. Regular meetings were arranged among all researchers throughout the process to progress discussion and finalisation of findings. The study results were then transformed and summarised into a common rubric. This rubric in Table 4 included; identified implementation models and framework, and results/themes of implementation approaches but not the outcomes of the intervention itself.

## Results

**Figure 1.**
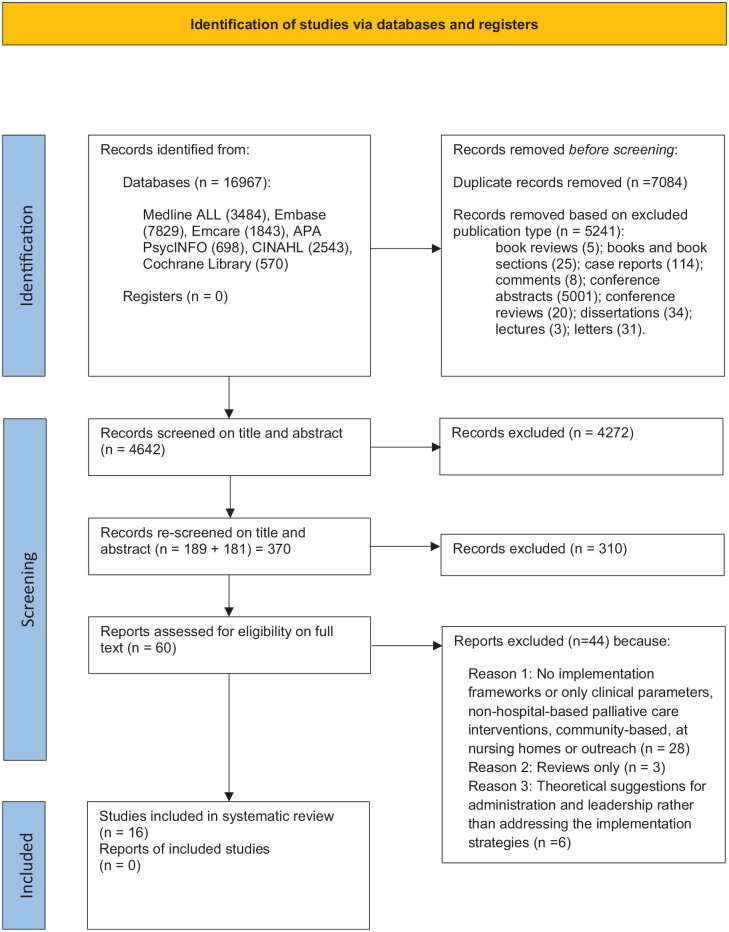
PRISMA 2020 flow diagram.

Following removal of duplicates and excluding publication types, 4642 records were screened on title and abstract. An additional update to the search was conducted via Medline Ovid 2021 to 2023 (current) and a further 181 records were screened on title and abstract, leading to a total of 60 records that were subject to full text review. Of these, 16 studies met the inclusion criteria (see Figure 1 for PRISMA flow chart).

### Quality assessment/risk-of-bias appraisal tools used to review studies

Study appraisal, also called validity assessment or quality assessment and critical appraisal pertains to a process of assessing the methodological quality of individual studies, by utilising the risk of bias validated tools.^15^ This is pertinent as it may affect both the results of the individual studies and ultimately the conclusions reached from the body of studies.

The following validated tools were used to assess quality, rigour and risk of bias; Critical Appraisal Skills Program (CASP) tool^23^ was used for critically appraising the qualitative studies, cohort studies and experimental/intervention studies, Mixed Method Assessment Tool (MMAT)^24^ for mixed methods studies and Joanna Briggs Institute (JBI)^25^ tool for pre-and-post (quasi-experimental) studies. Another risk of bias tool^26^ was used for the quantitative cross-sectional studies to assess 10 characteristics of the study. Quality assessment was based on the implementation data provided, rather than the overall study data. Qualitative studies were given a ranking of 1 to 10, cohort studies were given a ranking of 1 to 12 and experimental studies were given a ranking of 0 to 11 based on how many of the CASP questions could be answered positively.^23,27^ The Mixed Method Assessment Tool was used with a ranking of 1 to 5 based on how many of the five Mixed Method Assessment Tool questions could be answered positively^24^ (see Table 2).

**Table 2.** Quality assessment/risk of bias appraisal tools used to review studies.

	Author name, journal	Critical appraisal tool	Study type	Rating	Quality
1	Tanzi et al.^28^ *BMC Palliative Care*	MMAT	Mix methods study	3/5	Medium
2	Van der Padt-Pruijsten et al.^29^ *Journal of Pain and Symptom Management*	JBI checklist	Pre-and-post design intervention study	9/9	High
3	Lally et al.^30^ *JCO Oncology Practice*	MMAT	Mix methods study	2/5	Low
4	Resick et al.^31^ *Journal of Palliative Medicine*	JBI checklist	Quasi-experimental intervention study	5/9	Medium
5	Zemplényi et al.^7^ *BMC Palliative Care*	CASP	Qualitative study	7/10	Medium
6	Lödel et al.^32^ *BMC Palliative Care*	Hoy et al.^26^ Risk of Bias Tool	Quantitative cross sectional	6/10	Medium
7	Zimmermann et al.^33^ *BMJ Supportive & Palliative Care*	CASP	Qualitative	8/10	High
8	Evans et al.^34^ *Psycho-Oncology*	MMAT	Mix methods study	3/5	Medium
9	DiMartino et al.^35^ *Healthcare*	Hoy et al.^26^ Risk of Bias Tool	Quantitative cross sectional	5/10	Medium
10	Zubkoff et al.^36^ *Palliative and Supportive Care*	Hoy et al.^26^ Risk of Bias tool	Cross sectional quantitative	9/10	High
11	Karim et al.^37^ *Journal of Oncology Practice*	CASP	Qualitative	7/10	Medium
12	Bristowe et al.^38^ *Palliative Medicine*	CASP	Qualitative	6/10	Medium
13	Einstein et al.^39^ *Journal of Oncology Practice*	JBI checklist	Quasi-experimental	6/9	Medium
14	Ileffe et al.^40^ *BMC Palliative Care*	JBI checklist	Pre-and-post design intervention study	5/9	Medium
15	Hannon et al.^41^ *The Oncologist*	CASP	Qualitative	7/10	Medium
16	Bakitas et al.^42^ *Palliative and Supportive Care*	CASP	Qualitative	8/10	High

### Study characteristics

The 16 included studies (Table 3) largely originated in high-income countries including the United States (US),^30,31,36,35,39,42^ Canada,^33,34,37,41^ United Kingdom (UK)^38^ and Europe including Hungary,^7^ Germany^32^ and Netherlands^29^ and Italy^28^ One study was conducted across multiple European countries^40^ (Table 3).

**Table 3.** Project and sociodemographic summaries.

Author name, journal	Research aim	Country/setting intervention target/focus	Study design and methodology
1. Tanzi et al.^28^ BMC Palliative Care	To evaluate a training course in PC complexity for health professionals belonging to tumour boards/multidisciplinary discussion groups and its impact on the appropriateness of referral to palliative care	Country/setting: A single, General hospital, which has a specialised Palliative Care Service in Italy Intervention: A training course in PC complexity for health professionals belonging to tumour boards, aimed to increase the ability of the clinical staff to assess palliative care needs. The training course (3 theoretical lessons in 3 afternoons) • Assessing the physical, psychological, social and spiritual symptoms. • Breaking bad news to patients and families • Sharing decision making with patients and families The trainees included physicians and nurses from the Hepatocarcinoma, Pancreatic, Ovarian and Lung tumour boards	A phase 0–II, mixed-method study, developed accordingly to the Medical Research Council framework for the assessment of complex interventions (quality assessment). Method: Data triangulation of: -professionals’ interviews /focus groups with tumour boards × 4 -evaluation of complexity of referred patients, before and after the intervention, using the PALCOM instrument (classifies patients as having high, medium, or low palliative complexity in need of basic or specialised palliative care.)
2. Van der Padt-Pruijsten et al.^29^ *Journal of Pain and Symptom Management*	Effect of implementation of a palliative care pathway on patients’ place of death and advance care planning	Country/Setting: A single hospital, the Netherlands (Departments of Oncology, Haematology and Lung diseases) Intervention: a ‘digital’ palliative care pathway’ developed by a multidisciplinary team (haematology, anaesthesiology, neurology, cardiology, 1 aged care, palliative care, hospital leaders). -the palliative care pathway is a structured electronic medical checklist (44 items) as per the Dutch and international guidelines for palliative care - 850 patients were included, 58% male	Prospective pre- and post-implementation study Method: - Purposive sampling - data collected from adult cancer patients treated in inpatient or outpatient clinics and who died within the specified time frame. - the palliative care pathway was started only at a median of 33 days before death
3. Lally et al.^30^ *JCO Oncology Practice*	To uncover barriers to palliative care referral. To develop and pilot a program for enhanced palliative care integration in oncologic care, the ‘Warm Handoff’. To iterate the Warm Handoff program through ongoing collaboration and mid-pilot feedback sessions.	Country/ Setting: A single, academic cancer centre USA Intervention: - Warm Handoff enabled referring oncologists to page a palliative care nurse, who would meet the patient, describe palliative care services, and help schedule a palliative care appointment. - Increased collaboration led to the creation of a clinical provider of the day care model, increasing capacity to see urgent consults. Target/focus: - seven oncologists and oncology nurse practitioners participated in the initial pilot	Mix methods study including surveys and qualitative feedback from respondents Methods: - Feedback on the pilot Warm Handoff Program was elicited in 3 ways: a. Formal survey (sent to oncologists via email) b. One-to-one feedback sessions between palliative care clinicians and referring oncologists to understand the latters’ needs c. A steering committee to identify barriers to effective integration and to develop responses.
4. Resick et al.^31^ *Journal of Palliative Medicine*	Developing and pilot-testing a nurse-led primary palliative care intervention for patients with advanced hematologic malignancies called SHARE: Supportive Care Management for Patients with Hematologic Cancers by Registered Nurses	Country/ Setting: Two academic hospitals, USA Intervention: - SHARE Intervention included monthly nurse led visits corresponding with regular oncology visits. - Flexibility of delivery (phone or face to face) and frequency of visits Target/focus: adults with recurrent or resistant haematologic malignancies, adult family-carers - 26 patients, 17 caregivers, 5 oncologists, and 2 practice nurses. Patients’ mean age = 62 years, 54% male, caregivers’ mean age = 63 years, 53% male	A single-arm pilot study of a nurse-led primary palliative care intervention Method: Phase 1: development of an oncology nurse-led primary palliative care intervention for patients with recurrent or resistant hematologic malignancies and their caregivers. Phase 2: evaluation of feasibility and acceptability -Patient and caregiver outcomes measured using validated instruments such as the Edmonton Symptom Assessment Scale, Hospital Anxiety Depression Scale, Distress Thermometer
5. Zemplényi et al.^7^ *BMC Palliative Care*	To give a comprehensive overview of the Palliative Care Consult Service program covering six components of the conceptual framework for integrated care in multi-morbidity (SELFIE; Sustainable intEgrated chronic care modeLs for multi-morbidity: delivery, FInancing, and performancE) To detail barriers and responses to Palliative Care Consult Service	Country/Setting: A single academic cancer centre in Hungary. 15 interviews with stakeholders (4 managers, 3 physicians, 4 non-physician HCP, 2 informal caregivers, 2 patients) Intervention: Palliative Care Consult Service program Target/ Focus: - program stakeholders - documents related to program	Qualitative; thick description used Method: -Document analysis performed (official documents of the program, grey literature, presentations and expert information) -Interviews conducted with purposive sample to understand views of palliative care consult service
6. Lödel et al.^32^ *BMC Palliative Care*	To evaluate the level of implementation of the 14 standard operating procedures for palliative care in the Comprehensive Cancer Centre network.	Country/setting: 16 cancer centres, Germany Intervention: -online-based survey on the implementation status, accessibility, practicality, utility and processes of these guidance documents. Target/focus: - 66 health care professionals (physicians, nurses) specialising in palliative care responded to the survey. Mean age = 41.9 years, females (56.1%)	Descriptive survey (online) Method: - online-based survey on the implementation status - survey data analysed descriptively
7. Evans et al.^34^ *Psycho-Oncology*	Integrating early palliative care into routine practice for patients with cancer: A mixed methods evaluation of the INTEGRATE Project	Country/Setting: Four hospital based cancer services, Canada Intervention: - The INTEGRATE Project was implemented and consisted of; a) interdisciplinary provider education and b) an integrated care model. Providers used the Surprise Question to identify patients for inclusion. -an integrated care model was adapted from the Gold Standards Framework Target/focus: - 4 primary care practices were included. The 4 cancer centres spanned rural and urban areas. - 114 healthcare providers including nurses, physicians, and allied health professionals - 760 patients with cancer (head and neck, glioblastoma, lung, gastrointestinal) were included. Mean age across hospitals: 65 years	A mixed methods study Method: - semi-structured interviews with care assessing INTEGRATE to understand provider attitudes and confidence for providing palliative care, use of palliative care tools and perceived barriers to delivering palliative care - self-administered web-based 20-item survey (Likert-type scales) administered pre-implementation and post-implementation
8. DiMartino et al.^35^ *Healthcare*	Using the Organisational Theory of Innovation Implementation to further understand the role of formal and informal implementation policies and practices as determinants of implementation effectiveness. To examine their role within the context of initiatives to increase palliative care consultation in inpatient oncology.	Country/setting: a single academic hospital, USA Intervention: - Two Triggered Palliative Care (TTPC) Consultations approaches on consistency and quality of consult implementation, operationalised as uptake and timeliness, on two distinct inpatient services (solid tumour medical and gynaecologic oncology) Target/focus: - A total of 9760 patient encounters	Mix methods study Methods: - data obtained on palliative care consults, linked to hospital use - Development of guidelines with criteria to initiate a palliative care consultation, supported by training in palliative care skills, communication and dedicated funds to service the palliative care consultations.
9. Zimmerman et al.^33^ *BMJ Supportive & Palliative Care*	Explaining the concept, systematic evaluation and components of a team-based outpatient early palliative care (TO-EPC) intervention	Country/Setting: Princess Margaret Cancer Centre, Canada Intervention: Early outpatient palliative care Target/ Focus: - 71 participants with advanced cancer	Grounded theory qualitative study of participants following phase II clinical cluster randomised trial Method: - Purposive sampling - Semi-structured interviews - TO-PEC intervention developed based on the Medical Research Council guidance on development of Complex Interventions
10. Bristowe et al.^38^ *Palliative Medicine*	To explore healthcare professionals’ perceptions of using a complex intervention (AMBER care bundle) to improve care for people approaching the end of life and their understanding of its purpose within clinical practice.	Country/ Setting: 3 acute tertiary hospitals, UK Intervention: In 2 hospitals, the AMBER bundle was implemented on all wards and in the third a stepwise implementation was piloted on 5 wards. Target/focus: - 20 interviews with health care professionals (HCPs) from 3 hospitals. - HCPs consisted of 6 junior nurses, 6 senior nurses, 4 junior doctors and 4 senior doctors, and 15 participants were females.	Qualitative study, semi-structured interviews of 20 HCPs Method: - Purposive sampling - Semi-structured interviews + inductive thematic analysis and informed by the Medical Research Council (MRC) guidance for process evaluations, focusing on context, *implementation of the intervention* and mechanism of impact.
11. Karim et al.^37^ *Journal of Oncology Practice*	Primary aim: to improve the rates of outpatient Goals of Care documentation. Secondary goals: to increase the proportion of patients who were seen by palliative care.	Country/Setting: academic, comprehensive cancer centre, Canada Intervention: Goals of Care (GOC) documentation and palliative care referral, standardised documentation system designed within the EMR. Target/Focus: - medical oncologists - patients with advanced breast, lung, colorectal or pancreatic cancer. - 303 unique patients with advanced cancer *62% male, median age 67 years.*	16-month quality improvement initiative at the Cancer Centre of South-Eastern Ontario Method: - Design based on the Define, Measure, Analyze, Improve, Control quality improvement methodology. - an ‘improvement team’ (study staff, palliative care physician, medical oncology personnel) to support Goals of Care documentation and support oncologists - patient medical records were monitored for GOC documentation - a number of quality improvement cycles with feedback provided.
12. Zubkoff et al.^36^ *Palliative and Supportive Care*	Developing a ‘toolkit’ to measure implementation of concurrent palliative care in rural community cancer centres	Country/Setting: Four hospital based cancer services in the rural setting, USA Intervention: Concurrent palliative care Target/Focus: stakeholders at rural cancer centres	Descriptive cross-sectional study Method: - Stakeholders asked to complete ‘toolkit’ for their centre. - The ENABLE RE-AIM Self-Assessment Tool includes 50 open and closed response items assessing ‘reach’ (21 items), ‘adoption’ (11 items), ‘implementation’ (14 items) and ‘maintenance’ (4 items)
13. Einstein et al.^39^ *Journal of Oncology Practice*	To evaluate the effects of an embedded palliative care model versus a usual-care model on quality end of life care metrics (use and timing of palliative care, location of death, hospice use)	Country/setting: An academic Oncology Clinic, USA Intervention: - embedding a palliative care team in the oncology clinic and identifying target patients with prespecified criteria. - patients could access palliative care either as (a) usual care at an outpatient clinic available on oncologist referral, or (b) through an embedded model involving palliative care co-location in cancer clinics and palliative care based on prespecified criteria rather than oncologist discretion Target/focus: - 114 patients (26 with access to the embedded model and 88 patients with access to usual care). - intervention group mean age = 67 years; 88% male and usual care group mean age = 63 years; 67% male.	Non-randomised clinical trial (Quasi Experimental) Method: - patients seen on a specific day accessed the embedded model, based on automatic criteria. - data abstracted from the Electronic medical record, including date of death, age at death + demographic information
14. Ileffe et al.^40^ *BMC Palliative Care*	The IMPACT project (Implementation of Quality Indicators in Palliative Care study) evaluated the quality indicators (Qis) as tools to improving palliative care for people with cancer or dementia in 5 European sites	Country/Setting: across hospital and community settings, across England, Germany, Italy, Norway and The Netherlands Intervention: - QIs and improvement strategies were developed for four types of settings (hospitals, care homes, hospices and community palliative care teams) - Quality Indicators used to prompt reflection, identification of areas of service for improvement and facilitate benchmarking Target/focus: - Health care professionals.	Pre-and-post-test study Method: Purposive sampling 1) Modelling palliative care services and selecting Quality Indicators (QI) to form an intervention 2) Case studies of intervention using the QI set supported by an expert in service change in general practice, community palliative care teams, care homes, hospital wards, in-patient hospices, with a pre-and-post evaluation 3) Outcome evaluation across settings - Participants in each setting were supported to identify ⩽3 QIs for focus. - The 32 QIs were derived from existing indicator sets and selected using a modified RAND Delphi procedure involving a mix of palliative care clinicians and researchers
15. Hannon et al.^41^ *The Oncologist.*	Primary aim: To investigate, from the perspectives of patients and caregivers, what the perceived role was of their oncologist (control and intervention groups of a randomised control trial) and of their palliative care physician (intervention group only). Secondary aim: To examine whether the perceived role of the oncologist differed between participants in the control and intervention groups.	Country/setting: 24 Medical oncology clinics, Canada Intervention: - to obtain the accounts and opinions of those patients who did or did not receive treatment from palliative care in the randomised control trial Target/focus: - patients’ diagnosis of stage IV various cancer types - estimated prognosis 6 months – 2 years and Eastern Cooperative Oncology Group performance status score of 0–2 - patients’ median age = 60.5 years	A qualitative study, nested in a randomised control trial Purposive sampling Method: - individual semi-structured interviews, data analyses in grounded theory approach. - the model consists of consultation and follow-up in the oncology palliative care clinic by a palliative care physician and nurse
16. Bakitas et al.^42^ *Palliative and Supportive Care*	Understanding oncology clinicians’ perspectives about the care of advanced cancer patients after completion of the ENABLE II (Educate, Nurture, Advise, Before Life Ends) randomised clinical trial (RCT) of a concurrent integrated oncology palliative care model	Country/Setting: A rural, tertiary care, academic National Cancer Institute-designated cancer centre, USA Intervention: ENABLE II – palliative care delivery Target/Focus: - 35 Oncology clinicians (radiation oncology physicians, nurse practitioners, oncology and haematology) interviewed - mean age = 48 years, 50% female	Qualitative study Method: - semi-structured interviews (a secondary qualitative study following an RCT), - thematic analyses

Studies designs included qualitative studies (*n* = 6 out of 16), descriptive/cross-sectional (*n* = 3), mixed-methods (*n* = 3), pre-and-post implementation designs (*n* = 2) and clinical/quasi-experimental studies (*n* = 2) studies.

Study participants included individuals (health professionals, patients) and the participating urban and rural oncology services centres (Table 3).

### Types of implementation frameworks

Seven studies satisfied all our criteria and utilised a validated implementation framework^7,28,33,36–38,42^ (Table 4). Of these, two studies included the implementation of a palliative care intervention (ENABLE RE-AIM Self-Assessment Tool for reach, effectiveness, adoption, implementation and maintenance)^36,43^ across four United States rural cancer services in hospitals.^36^ One study employed a quality improvement framework based on domains of Define, Measure, Analyze, and Improve to implement an intervention aiming to increase documentation of goals of care and secondarily, palliative care referral.^37^ Three studies utilised the Medical Research Council (MRC) guidance for process evaluation, focusing on context, application of the intervention, mechanism of impact^38^ and evaluation phases when establishing hospital based palliative care services^28,33^ (Table 4). A study in Hungary used the SELFIE implementation model (Sustainable integrated chronic care models for multi-morbidity: delivering, financing, and performance) to guide their review of a palliative care consultation service programme^7^ (Table 4). This framework organises elements of integrated care into the six World Health Organisation components of health systems (leadership and governance, service delivery, information and research, technologies and medical products, financing and workforce).^7^

**Table 4.** Implementation models, frameworks and emerging themes/results.

Implementation models and frameworks		Implementation strategies adopted (NOT outcomes of the intervention itself)
1. Tanzi et al.^28^ *BMC Palliative Care*	Yes: Medical Research Council (MRC) guidance on complex intervention design Moore model was used as the evaluation framework, comprising five orders of learning, from attendance (Level 1) to change in practice performance (Level 5). -The study evaluated the impact of the training related to: • Increasing palliative care competencies regarding complexity (Moore Level 3) • Evaluation of participants’ performances in addressing more complex palliative care patients to specialised service (Moore Levels 4 and 5).	Four teams of professionals were trained in the palliative care educational intervention which included adaption of information technology systems, a training course, and bedside training for all the health professionals involved in multidisciplinary pancreas, lung, ovarian and liver tumour boards. The training, adapted to trainees’ needs and observations also led to organisational modifications.
2. Zemplenyi et al.^7^ *BMC Palliative Care*	Yes: SELFIE; Sustainable intEgrated chronic care modeLs for multi-morbidity: delivering, FInancing and performancE. This framework organises elements of integrated care into the 6 World Health Organisation components of health systems (leadership and governance, service delivery, information and research, technologies and medical products, financing, workforce).	Service delivery: Continued outpatient palliative care follow up post discharge from hospital. Leadership & Governance: Management of the Clinical Centre was very supportive of the implementation. Workforce: New roles were created to support the provision of the program, including palliative care coordinator. Financing: Despite the lack of reimbursement from the National Healthcare Fund, the Palliative Care Consultancy Service program could be funded from a variety of sources. Technologies and Medical Products: The hospital information system was changed to support the palliative care requests and documentation within the clinical centre. Information & Research: The program conducted annual retrospective anonymous analyses of Palliative Care Consultancy Service activities.
3. Zimmerman et al.^33^ *BMJ Supportive & Palliative Care*	Yes: Medical Research Council guidance on developing and evaluating complex interventions with evaluation and implementation phase underpinning this study.	Emphasis on early access to palliative care, provision of palliative care in collaboration with other treating healthcare teams Provision of palliative care based on the needs of the patient and family, rather than on prognosis.
4. Zubkoff et al.^36^ *Palliative and Supportive Care*	Yes: RE-AIM (reach, effectiveness, adoption, implementation, and maintenance)	The study developed 4 instruments to measure ENABLE Implementation: • The ENABLE RE-AIM Self-Assessment Tool to assess reach, adoption, implementation, and Maintenance • The ENABLE General Organisational Index to assess institutional implementation • The Implementation Costs Tool • The Oncology Clinicians’ Perceptions of Early Concurrent Oncology palliative care survey
5. Karim et al.^37^ *Journal of Oncology Practice*	Yes: The Define, Measure, Analyze, Improve, Control Quality Improvement methodology.	A number of palliative care implementation improvement cycles consisted of: • Selecting appropriate patients • Teaching physicians how to fill out needed forms and encouraging them to complete a form any time an outpatient conversation occurred • Training medical records clerks to recognise, scan and upload forms onto the electronic patient record • Ensuring electronic pharmacy records were being used to send e-reminders to medical oncologists
6. Bristowe et al.^38^ *Palliative Medicine*	Yes: Medical Research Council guidance on developing and evaluating complex interventions AMBER care bundle = the complex intervention	Strategies to identify and reach the target population included a consideration for hospital-wide ward composition and casemix which could impact upon use of the intervention implementation Utilising appropriate methods of implementation after understanding how complex interventions towards End-of-Life (EOL) are perceived, interpreted and then acted upon and by whom Participants described challenges with implementation due to reduced familiarity and limited exposure to the palliative care intervention in wards where few patients were appropriate for the intervention
7. Bakitas et al.^42^ *Palliative and Supportive Care*	Yes: ENABLE palliative care	Multidisciplinary approach, appropriate timing and patient readiness Additional resources including time by the palliative care team to assist in care of medically and socially complex patients and families.
No formal Implementation Framework cited, but report use of some implementation strategies		
8. Van der Padt-Pruijsten et al.^29^ *Journal of Pain and Symptom Management*	No Implementation Framework	In the last 90 days of life, a pain management team and a specialised palliative care team was consulted in the pre- and post-period. Awareness among healthcare staff created by extensive education on how to use the palliative care pathway End-of-life discussions and shifting to symptom-centred care goals associated with less utilisation of anticancer treatment and radiotherapy
9. Lally et al.^30^ *JCO Oncology Practice*	No Implementation Framework The Warm Handoff Program/Process The Clinical Provider of the Day (CPOD) Care Model	A steering committee, consisting of oncologists from the Division of Women’s Cancers, four palliative care physicians and nurse practitioners, two nurses and two administrators from palliative care sought to identify barriers to effective integration and to brainstorm solutions to enhance collaboration and care. Surveys via email to ask the referring clinicians about their experiences with the Warm Handoff and palliative care in general A palliative care physician and nurse practitioner contacted participating breast and gynaecology oncologists to elicit feedback and to understand the reasons for calls to the Warm Handoff pager. New patients to palliative care referred from the breast and gynaecologic oncology group were tracked using Tableau, a dashboard developed at Dana-Farber Cancer Institute. The dashboard presented data from the electronic medical record, including new patient visits over time and primary disease centre to which the patient was registered.
10. Resick et al.^31^ *Journal of Palliative Medicine*	No Implementation Framework SHARE used as a model and nurse-led intervention, which is a different model of care provision.	Training of two oncology nurses as SHARE nurse interventionists with the goal of enrolling patients with advanced hematologic malignancies and their caregivers. Intervention protocol developed in Phase 1, followed by incorporation of Phase 1 patient, caregiver, and clinician feedback into Phase 2, which assessed intervention feasibility and acceptability. Three-day training managed by palliative care and nursing education experts included didactic and interactive observed practice sessions using hematologic malignancy case studies and standardised patients. Study staff met regularly with the nurse interventionist to identify eligible patients from upcoming clinic schedules. Intervention was integrated into research nurse’s regular workload, with ongoing coaching and support from the nurse research coordinator.
11. Lödel et al.^32^ *BMC Palliative Care*	No Implementation Framework The Standard Operating Procedures (SOPs) were in essence the intervention, with implementation measured by the frequency of use of these in routine clinical practice.	Standard Operating Procedure (SOP) awareness was created mostly by departmental team meetings, ensuring that more than half of respondents were aware that they were freely available on the Comprehensive Care Centres network website. Survey for measures that could facilitate the integration and use of Standard Operating Procedures in clinical practice, measure for quality assurance and improvement in palliative care An expert committee consisting of representatives from the 16 clinical sites at a meeting of the working group on palliative medicine developed a catalogue of measures aimed at increasing the awareness and use of SOPs. Creating a wish list (sound board) for respondents’ ideas for further Standard Operating Procedures Barriers to SOP implementation included ‘difficulty findings the SOPs’, the perception that they had ‘no practicality in everyday life’, ‘lack of time’ and ‘difficulty to change common routines’ and ‘lack of integration into clinical routine’. Facilitators for SOPs implementation included internal activities such as; • Training by colleagues or discussion in a multi-professional team to promote awareness of the SOPs • Linking of SOPs with the request for and advice of palliative care support team within the hospital information system
12. Evans et al.^34^ *Psycho-Oncology*	No Implementation Framework An integrated care model was developed by a group including providers, administrators and patient and family advisors	Co-design of the intervention by improving provider confidence to deliver palliative care and to initiate the Advanced Care Planning conversation. The model was embedded into existing workflows, electronic systems and initiatives. Time, funds and human resources were dedicated to Implementation. Senior managers provided visible support and clear parameters for the model. Importance of partnerships with external providers and organisations were highlighted.
13. DiMartino et al.^35^ *Healthcare*	No Implementation Framework	Two ‘Triggered palliative care consultation’ (TPCC) approaches were used to promote palliative care consult implementation in the gynaecologic and medical oncology services. TPCC supported by a single strategy in gynaecologic oncology included one-page written guideline of clinical criteria for initiating a consult (i.e., uncontrolled pain, nausea or vomiting, malignant bowel obstruction, frequent readmissions, request for hospice, or resistance to advanced care planning). TPCC supported by multiple strategies in medical oncology included; • Training of residents in palliative care skills of advanced care planning communication • Clinician prompting of eligible patients by palliative care service • Medical chart review to identify patients with advanced cancer (i.e., metastatic or uncontrolled symptoms) • Dedicated institutional funding • Appointed champions from palliative care
14. Einstein et al.^39^ *Journal of Oncology Practice*	No Implementation Framework Embedded palliative care model described briefly in article, but further details of this specific model available in prior publication by same author (see Einstein et al.^39^).	Two palliative care providers (physician and chaplain) were accommodated in the oncology intravenous therapy clinic, sharing the workroom and (often) seeing patients concurrently with the oncologists Patients in the embedded care model were significantly more likely than those in the usual-care model to have their first encounter as an outpatient versus inpatient (*p* = 0.003) Balancing act between enhancing palliative care referrals for increasing patients’ access to palliative care, and conserving palliative care resources including palliative care staff as a key consideration when implementing a new service
15. Ileffe et al.^40^ *BMC Palliative Care*	No Implementation Framework A qualitative description of palliative care models in Europe informed developmental strategies to improve the organisation of palliative care using Qis The IMPACT project (Implementation of Quality Indicators in palliative care study), informed by the UK Medical Research Council (MRC) framework	Assessment of acceptability and feasibility important for successful implementation. The quality indicators (QI) set was too specialist focused (in palliative care terms) and not useful enough for non-specialists (care homes and in general practice) The top-down engagement was difficult in implementation Staff changes, funding difficulties and competing organisational demands, delaying data transfer on outcomes to the IMPACT research team. Establishing a research and development culture of the organisation before implementation The IMPACT project selected appropriate quality indicators using a modified RAND Delphi procedure, resulting in 32 quality indicators which may be used to evaluate the outcomes of service implementation going forward
16. Hannon et al.^41^ *The Oncologist*	No Implementation Framework Model of integrating palliative care into oncology care by early intervention in a specialised clinic setting. This study was a secondary qualitative piece of research after a cluster randomised control trial for early palliative care integration.	palliative care and oncology (discrete and complimentary roles), supporting a model of integrating palliative care into oncology care by early intervention in a specialised clinic setting Providing access to palliative care for participants for symptom management and psychosocial support meant their oncologist could focus on disease-specific issues and cancer treatment The integrated care model allowed timely, efficient, and coordinated management of multiple physical, psychosocial, and existential needs by an interdisciplinary team Using integrated care model compared to ‘solo practice model’ in which oncologists assumed all care from diagnosis to death, and the ‘congress practice model’ referring to multiple consultants who specialised in various symptom and psychosocial concerns

A further nine studies included components of implementation science to report upon outcomes of interventions for integration of palliative care in oncology settings but did not cite a specific implementation framework. These studies were establishing different integrated palliative care models, for example, a digital ‘palliative care pathway’ developed by the multidisciplinary team,^29^ and a nurse-led primary palliative care implementation programme for patients with advanced hematologic malignancies^31^ (Table 4). Strategies used and reported by these nine studies which aligned with implementation domains included: strategies to identify and reach the target population^29,39^; relationship building and engagement^30^; co-design of the intervention^34^; training and education^32^; attention to workplace culture and practices^34,39^ and assessment of acceptability and feasibility.^30,31,40^

### Synthesis of enablers and barriers reported in palliative care implementation studies in cancer services

The details of the implementation frameworks for the included studies along with the study descriptions, and the nature of the evaluations undertaken are provided in Table 4. The key findings of enablers and barriers from our included studies with more detailed explanation have been grouped based on: a) staff; b) patients; c) system and d) implementation guidelines.

### Staff enablers and barriers

**• Dedicated staff**

The availability of dedicated palliative care specialist staff was key to the success of programs,^36^ including the ability to respond to urgent consults and the availability of clinicians to discuss challenging cases.^30^ A designated ‘clinical provider of the day’ programme allowed for a noteworthy increase in the capacity to see urgent palliative care consults with an average of 19 patients seen per month.^30^

**• Clearly defined roles**

There was a generalised perception amongst oncologists that the roles of palliative care clinicians were as ‘co-managers’ and ‘consultants’^42^ with the need for clarity emphasised around the roles and responsibilities of clinicians.^34^ Cancer-directed treatments were perceived to be the main domain of the oncologist, while pain and symptom control, and care for the patient and their carer were identified as the primary role of palliative care.^41^ A nurse led supportive care clinic with clearly defined roles was set up for patients with haematological cancer which was widely supported by service oncologists, 75% of whom agreed it improved the quality of patient care.^31^

**• Health care providers’ confidence**

Integrating early palliative care into routine care for patients with advanced cancer led to an improvement in oncologists’ confidence to deliver palliative care^34^ and to initiate the Advanced Care Planning conversation with patients.^28,42^ Nevertheless, some physicians expressed doubts about starting discussions with patients as a result of uncertainty regarding prognosis and unknown consequences for the physician−patient relationship.^29^ This prognostic uncertainty was particularly cited as a barrier in the setting of haematological cancers.^31^

### Patient enablers and barriers

**• Patient perceptions**

Patients and caregivers were largely satisfied (84%) with the palliative care intervention^31^ and described it as providing: swift and personalised symptom management; holistic support for patients and caregivers^41^; guidance in decision-making and preparation for the future^28,37^ and assisting the caregivers with understanding their family member’s illness and cancer ‘journey’.^31,42^ However, potential barriers included the uncertain course of hematologic malignancies and low levels of patient interest in palliative care research (89%) with few patients completing surveys reporting feedback about palliative care.^31^ The stigma surrounding the name ‘palliative’ was reported as a barrier to early palliative care referral that needed to be explicitly addressed within the intervention programme.^33^

### System enablers and barriers

**• Incorporation into existing workflow**

The integrated palliative care model was most effective when it was implanted into existing workflows and supported by electronic systems.^34^ Conversely, challenges to implementation included a lack of clinician readiness at the clinical site was observed as an implementation challenge^36^ and practices that involved non-palliative care clinicians being tasked with additional clinical responsibilities such as completing symptom assessment tools.^40^ Others noted barriers included lack of acceptability and feasibility, with work tools that were considered too specialist focused^40^ or not practical, difficult to locate, time consuming and poorly integrated into routine workflows and routines.^32^

**• Administrative barriers (time, funds and human resources)**

Administrative difficulties included securing optimum space, staff, and sufficient time to launch the implementation programme.^36^ Authors of early palliative care intervention studies described potential cultural or professional conflicts for implementing a complex intervention in clinical settings under the care of multiple specialities.^38^

The integrated palliative care model was most successful if the senior managers and heads of tumour streams provided support,^28^ and time, funds, education and human resources were dedicated to the implementation programme.^31,34,38^ An important provision is that a balance must be struck between broadening palliative care referral criteria for increasing patient access to palliative care,^28^ and taxing limited available palliative care resources including workforce shortages and space constraints.^39^ A lack of a standardised Institutional Review Board approach including their hesitancy to approve palliative care research and quality audit activities was encountered by the researchers when undertaking evaluation of outcomes.^36^

**• Rapid staff turnover**

The rapid turnover of staff in the hospital/clinical settings was reported as having a detrimental impact upon implementing a new service intervention, resulting in delays and funding difficulties with ongoing requirements for staff training,^31,40^ disrupted data collection, clinical relationships and lines of communication.^31,40^

**• Organisation culture (Training/Health Education)**

Within the organisation, the role of a multidisciplinary team^28^ was recognised to be pertinent in the delivery of palliative care interventions.^38,41^ Considering those studies involving education interventions aimed at increasing clinician ability to recognise palliative care complexity,^28,42^ the role of a multidisciplinary team was regarded positively and facilitated earlier access to the palliative care team.

Others noted positive organisational change available in early palliative care through the AMBER study which provided a tool to facilitate change to the way care was delivered and also facilitated change to the culture of care delivery.^38^ Potential facilitators for palliative care implementation included internal activities such as intercollegiate palliative care training or multidisciplinary team discussions to promote awareness of tools to assist palliative care provision.^28,32^

**• Recognition of champions**

The value of clinical advocates and champions was highlighted as supporting a successful palliative care programme implementation.^34^ To meet the patient and clinician needs, a strong collaboration between palliative care and oncology teams was necessary.^30^

**• Communicating**

The oncologists viewed the role of integrated palliative care as facilitating a comprehensive approach to treating the patient with an advanced cancer, and palliative care consultants were seen as sharing the load.^28,42^ The importance of ‘timing’ and ‘time’ was emphasised for the palliative care conversations and oncologists involved palliative care clinicians early on in order to establish good communication enabling end-of-life treatment planning for a patient with advanced cancer.^42^ However, many haematologists felt the unpredictable nature of hematologic malignancies made it difficult to identify the appropriate time to introduce palliative care consultations.^31^ Hence, there were a few missed opportunities where palliative care could not be introduced.^42^

Physicians conveyed doubts about starting discussions with patients around palliative care as a result of prognostic uncertainty and concerns about destroying patients’ hopes and negatively affecting the physician−patient relationship.^29^ Training in having such discussions was identified as an important aspect of doctor-patient communication.^28,34^

### Implementation guidelines

The maintaining of fidelity and consistency of the intervention across individual patients and different sites required ongoing training and education of staff.^28–30,34,38,39^ A number of authors cited the lack of relevant health policy regulations and financial schemes to support palliative care services. They highlighted the need for distinction on an institutional level between feasibility and ongoing sustainability, which required a more systematic approach.^7,28,33,36,41^ Furthermore, the divergent levels of development across services required flexibility in the methods for service engagement and the setting of benchmarks to assess success.^32,35,40^

In order to demonstrate the application of implementation frameworks to this field, the enablers and barriers for palliative care implementation identified through the narrative synthesis are presented in Table 5, and grouped into categories using the example of the RE-AIM Frame-work (Reach, Effectiveness, Adoption, Implementation and Maintenance).^44^

**Table 5.** Narrative synthesis of enablers and barriers for palliative care implementation under RE-AIM Framework.

	Those factors with an impact categorised according to the domains of RE-AIM	
	Enablers	Barriers
Reach	• A model of early integrated palliative care was developed by a group including providers, administrators, and patient and family advisors (Evans et al.^34^) • Maintaining good physician-patient relationships to address early integration of palliative care and quality for end of life in patients with advanced cancer (Hannon et al.^41^) and supporting palliative care conversations between doctors and patients (Bakitas et al.^42^; Evans et al.^34^; Hannon et al.^41^)	• Lack of awareness for palliative care communication for healthcare providers, patients and public (van der Padt-Pruijsten et al.^29^; Zimmermann et al. 2019)^33^ • Lack of involvement of a multidisciplinary team (van der Padt-Pruijsten et al.^29^)
Effectiveness	• Presence of dedicated programme staff and palliative care specialists (Lally et al.^30^; Resick et al.^31^; Zubkoff et al.,^36^ Tanzi et al.^28^) • Three-day training and workshops led by palliative care and nursing education experts included didactic and interactive practice sessions (Resick et al.^31^) • Palliative care and Oncology integrated model allowed for timely, efficient, and coordinated management of multiple physical, psychosocial, and existential needs of patients by an interdisciplinary team (Bakitas et al.^42^; Hannon et al.^41^, Tanzi et al.^28^) • Increased patients’ satisfaction with the oncologist, care and cancer treatment (Hannon et al.^41^; Zimmermann et al.^33^) • Co-design of the intervention incorporating patient, caregiver and provider feedback (Resick et al.^31^; Evans et al.^34^), resulted in improved • Provider confidence to deliver palliative care and to initiate the Advanced Care Planning conversation. (Evans et al.^34^) • Intervention embedded into existing workflows and electronic systems (Evans et al.^34^) • Intervention that facilitated a multidisciplinary approach, appropriate timing and patient readiness, additional resources including of time by the palliative care team to assist in care of medically and socially complex patients and families were found to be most effective (Bakitas et al.^42^)	• Communication barriers between the physicians and patients to talking about the end of life and palliative care (Zimmermann et al.^33^) • Physicians’ perceptions of what is required for maintaining optimum doctor-patient relationship sometimes precluded communication about early palliative care integration and discussions about End of Life care (Zimmermann et al.^33^) • Stigma surrounding the name ‘palliative care’ (Zimmermann et al.^33^) • Administrative barriers impacted fidelity and consistency of the palliative care intervention. These included reduced familiarity and limited exposure to the palliative care intervention in wards where few patients were appropriate for the intervention (Bristowe et al.^38^). • Lack of time for healthcare staff and clinicians (DiMartino et al.^35^) • Professional/organisational cultural barriers for implementing a palliative care intervention in hospital-wide ward which catered for patients under the care of multiple different specialties (Bristowe et al.^38^) • Lack of training for palliative care team (Evans et al.^34^, Tanzi et al.^28^) • Lack of defining clear roles (Evans et al.^34^)
Adoption	• Community Advisory Groups (Evans et al.^34^) to oversee monitoring and evaluation of the palliative care intervention programme (DiMartino et al.^35^) • Use of co-design with key clinical partners to develop intervention (Evans et al.^34^) • Gathering real-time qualitative feedback from the oncologists by the study coordinators with responses and adaptation of the intervention facilitated uptake (Karim et al.^37^; Zimmermann et al. 2016) • Palliative care ‘embedded’ into systems of cancer care (Einstein et al.^39^) • Culture of ‘readiness’ assessed via surveys and systems and practices of workflow understood prior to service change (Bakitas et al.^42^; Zubkoff et al.^36^) • Development of materials and resources to facilitate the intervention delivery (Bakitas et al.^42^) • The SELFIE Framework (Zemplényi et al.^7^) helped in palliative care adoption by following up patients post discharge from hospital, creating new roles (funded) to support the provision of the program, and providing regular feedback for adaptation of intervention for uptake	• Physicians’ perceptions as many continue to equate palliative care with End of Life care (van der Padt-Pruijsten et al.^29^; Zimmermann et al. 2016) • Intervention delivery disrupts routine workflow and usual clinical practice (Lödel et al.^32^)
Implementation	• Integrated palliative care merged in existing workflows, electronic systems and a network of dedicated staff (Evans et al.^34^; Resick et al.^31^) • Committed time, funds and human resources (Evans et al.^34^; Resick et al.^31^) • The training intervention for health care staff elicited new adjustments to the clinical care pathway of the tumour boards (Tanzi et al.^28^). • Clear roles and responsibilities of clinicians (oncologists and palliative care physicians) (Bakitas et al.^42^) • Quality indicators for implementation (Ileffe et al.^40^) • Linking palliative care team into hospital information system (HIS) (Lödel et al.^32^; Zemplényi et al.^7^) • Documenting goals of care documentation rates post-implementation (Karim et al.^37^)	• Lack of sustained relevant implementation strategies and guidelines (Hannon et al.^41^; Zemplényi et al.^7^; Zimmermann et al.^33^; Zubkoff et al.^36^) • Lack of awareness of multiple formal implementation policies (DiMartino et al.^35^) • Difficulty in access, relevance of guiding documents, lack of time, and difficulty to change common routines and lack of integration of guidance into clinical routine (Lödel et al.^32^) • Absence of a standardised Institutional Review Board approach to facilitate implementation evaluation (Zubkoff et al.^36^) • Rapid staff turnover (Ileffe et al.^40^; Resick et al.^31^) • Difficulty maintaining fidelity + consistency of the intervention across individual patients and different sites (Bristowe et al.^38^; Einstein et al.^39^; Evans et al.^34^; Lally et al.^30^; Van der Padt-Pruijsten et al.^29^)
Maintenance	• Balancing palliative care referral with the limited available staff members (Einstein et al.^39^) • Recognising and affirming clinical champions (Evans et al.^34^)	• Lack of relevant health policy regulations and financial schemes to support palliative care services (Hannon et al.^41^; Zemplényi et al.^7^; Zimmermann et al.^33^; Zubkoff et al.^36^) • Lack of established, clear benchmark criteria to assess palliative care implementation and impact (Bristowe et al.^38^; DiMartino et al.^35^; Einstein et al.^39^; Evans et al.^34^; Ileffe et al.^40^; Lally et al.^30^; Lödel et al.^32^; Van der Padt-Pruijsten et al.^29^)

## Discussion

This systematic review supports the need to translate the strong evidence-base supporting integrated palliative care into oncology practice by examining the rigour with which implementation science principles have been employed.

**i) Main findings/results of the study**

This review indicates that many researchers have hinted at components of implementation frameworks without explicitly using them when establishing integrated palliative care in their hospital settings. This finding is consistent with observations made in the implementation science literature about the paucity of explicitly naming frameworks.^45,46^ Those studies that clearly utilise implementation frameworks to formally address the issues related to system, staff, patients and intervention have largely been published in recent years, suggesting this is an emerging area in the field.^12^

The few studies in this review which have utilised implementation frameworks to guide service delivery have variously used RE-AIM (Reach, Effectiveness, Adoption, Implementation and Maintenance) framework,^36,42^ SELFIE model (Sustainable integrated chronic care models for multi-morbidity: delivering, financing and performance),^7^ MRC (Medical Research Council) framework to guide complex interventions,^28,33,38^, and the quality improvement approach of Define, Measure, Analyze and Improve.^37^ The increase of studies in the recent past which have utilised implementation frameworks may suggest that it is an emerging area in the field of palliative care integration.

**ii) What this study adds:**

This review utilises multiple, validated tools for included studies to assess the quality, rigour and risk of bias as part of quality appraisal, with medium scoring allocated to most studies.^23–26^ The quality appraisal ranking was high for four studies, medium for 11 studies, and low for one study.

While the evidence-base underpinning early integration of palliative care is now mature, it appears that the evidence guiding implementation into practice is less developed. The effective utility of the implementation frameworks for systematically implementing palliative care in hospital clinical settings has been shown in this systematic review with narrative synthesis as well as by previous studies conducted in other settings (such as community settings and aged care facilities) and with other populations such as non-oncology (chronic disease) cohorts and chronic asthma sufferers.^47,48^

We posit the use of an implementation framework such as RE-AIM should be considered going forward to systematically present the barriers and enablers in palliative care implementation programs across hospitals in the oncology units. Having a systematic approach to the implementation of a new service or a new aspect of a service will make explicit not only of those areas which have facilitated the success of the project, but also enable a clearer understanding of reasons underpinning failure to make a change. This in turn may help stakeholders including policy makers and service providers in allocating precious resources towards planning and systematically implementing integrated palliative care programs in the hospital-based cancer services.

The systematic approach offered by implementation science frameworks is also key to ensuring the sustainability of those successfully established programs. Importantly, having a common language to report these experiences of service implementation such as those constructs of RE-AIM framework will allow the learning to be available to other services considering establishing integrated palliative care programs.

**iii) Strengths and weaknesses/limitations of the study:**

There are few limitations to this review. While care was taken in screening studies, it is possible that some studies were missed, as implementation methodologies were not consistently reported. Only the studies written in English language were selected, and these were conducted in high-income countries, so the implementation strategies presented may not be applicable in the low-to-middle-income countries. Some qualitative studies did not provide adequate details about the interview methodologies, or justification for observing rigour and reflexivity and so were omitted, as was the considerable grey literature and unpublished work in this area. However, the strengths of this review include a robust and systematic screening process, and using multiple, validated risk-of-bias tools for quality appraisal of various study designs, to have a holistic view of the palliative care implementation strategies.

The type of Implementation framework that a researcher may want to select could be affected due to the diverse interventions (nurse led, education training, collaborative) and settings for the studies. However, using a formal implementation framework prompts comprehensive consideration of the different areas of influence, of activity and evaluation, ensuring a systematic approach to the programme development is available.

## Conclusion

There is very limited utilisation of implementation frameworks to integrate early palliative care into hospital oncology services. Yet the multidimensional, system-wide approaches within implementation science frameworks offer a structure to guide and evaluate programme development. Key elements of implementation to consider when establishing palliative care service integration in cancer centres include a focus on co-design of the intervention and processes of implementation, staff education and training including around processes, communication and introducing referrals, dedicated resources, recognition of champions and more effective communication across the multidisciplinary teams in the hospital. The opportunities offered by implementation science to guide the effective integration of palliative care into cancer care suggest that the formal use of implementation frameworks should be adopted in future service development initiatives.

## Supplemental Material

sj-pdf-1-pmj-10.1177_02692163231186177 – Supplemental material for An evidence-base for the implementation of hospital-based palliative care programs in routine cancer practice: A systematic review Click here for additional data file. Supplemental material, sj-pdf-1-pmj-10.1177_02692163231186177 for An evidence-base for the implementation of hospital-based palliative care programs in routine cancer practice: A systematic review by Farwa Rizvi, Helen Elizabeth Wilding, Nicole M Rankin, Roslyn Le Gautier, Lorna Gurren, Vijaya Sundararajan, Kylee Bellingham, Joyce Chua, Gregory B Crawford, Anna K Nowak, Brian Le, Geoff Mitchell, Sue-Anne McLachlan, Tanara Vieira Sousa, Robyn Hudson, Maarten IJzerman, Anna Collins and Jennifer Philip in Palliative Medicine

sj-pdf-2-pmj-10.1177_02692163231186177 – Supplemental material for An evidence-base for the implementation of hospital-based palliative care programs in routine cancer practice: A systematic review Click here for additional data file. Supplemental material, sj-pdf-2-pmj-10.1177_02692163231186177 for An evidence-base for the implementation of hospital-based palliative care programs in routine cancer practice: A systematic review by Farwa Rizvi, Helen Elizabeth Wilding, Nicole M Rankin, Roslyn Le Gautier, Lorna Gurren, Vijaya Sundararajan, Kylee Bellingham, Joyce Chua, Gregory B Crawford, Anna K Nowak, Brian Le, Geoff Mitchell, Sue-Anne McLachlan, Tanara Vieira Sousa, Robyn Hudson, Maarten IJzerman, Anna Collins and Jennifer Philip in Palliative Medicine
