# Supplementary material for: An evidence-base for the implementation of hospital-based palliative care programs in routine cancer practice: A systematic review
Source: Palliat Med. 2023 Jul 8;37(9):1326–44. doi: 10.1177/02692163231186177 (PMC10548767; doi:10.1177/02692163231186177)
Supplement: sj-pdf-1-pmj-10.1177_02692163231186177 – Supplemental material for An evidence-base for the implementation of hospital-based palliative care programs in routine cancer practice: A systematic review [file sj-pdf-1-pmj-10.1177_02692163231186177.pdf]

Supplementary File 1: Search strategy for Ovid MEDLINE(R) ALL 1946 to March 10, 2021

1. Palliative Care/ or Palliative Medicine/ or "Hospice and Palliative Care Nursing"/ or Hospices/ or Hospice Care/
2. (palliative or hospice* or "end of life care").ti,ab.
3. 1 or 2
4. exp Neoplasms/
5. exp Medical Oncology/
6. exp Oncologists/ or Oncology Nursing/
7. Cancer Care Facilities/ or Oncology Service, Hospital/
8. (cancer* or carcinoma* or leukaemia* or leukemia* or lymphoma* or malignan* or melanoma* or neoplasia* or neoplasm* or oncolog* or tumor* or tumour*).ti,ab.
9. 4 or 5 or 6 or 7 or 8
10. Cancer Care Facilities/ or Oncology Service, Hospital/
11. exp Hospitals/
12. exp Hospital Administration/
13. inpatients/ or outpatients/ or exp Hospitalization/ or Home Care Services, Hospital-Based/
14. (hospital* or inpatient* or outpatient* or tertiary care or ward* or cancer care facilit* or cancer care unit* or cancer cent*).ti,ab.
15. 10 or 11 or 12 or 13 or 14
16. implementation science/ or (implement* or introduc*).ti,ab.
17. establish*.ti. or establishment.ti,ab.
18. "diffusion of innovation"/ or innovat*.ti.
19. Quality Improvement/ or quality improvement.ti,ab.
20. Health Plan Implementation/
21. "Delivery of Health Care, Integrated"/ or integrat*.ti,ab.
22. Program Development/ or development.ti,ab. or develop*.ti.
23. exp Evidence-Based Practice/
24. access*.ti. or (increase access* or early access).ti,ab.
25. (utilis* or utiliz*).ti.
26. *interdisciplinary communication/
27. Pilot Projects/ or Feasibility Studies/ or (pilot or feasibility).ti.
28. Perception/ or "Attitude of Health Personnel"/ or attitude*.ti.
29. "Referral and Consultation"/ or (refer* or consult* or early palliative).ti.
30. program evaluation/
31. (pathway* or barrier* or enable*).ti.
32. 16 or 17 or 18 or 19 or 20 or 21 or 22 or 23 or 24 or 25 or 26 or 27 or 28 or 29 or 30 or 31
33. 3 and 9 and 15 and 32
34. (child* or infant* or paediat* or pediat* or adolescen* or young or youth).ti.
35. 33 not 34
36. limit 35 to English language

Supplementary File 2: Search strategies for all databases

Ovid MEDLINE(R) ALL 1946 to March 10, 2021

1. Palliative Care/ or Palliative Medicine/ or "Hospice and Palliative Care Nursing"/ or Hospices/ or Hospice Care/
2. (palliative or hospice* or "end of life care").ti,ab.
3. 1 or 2
4. exp Neoplasms/
5. exp Medical Oncology/
6. exp Oncologists/ or Oncology Nursing/
7. Cancer Care Facilities/ or Oncology Service, Hospital/
8. (cancer* or carcinoma* or leukaemia* or leukemia* or lymphoma* or malignan* or melanoma* or neoplasia* or neoplasm* or oncolog* or tumor* or tumour*).ti,ab.
9. 4 or 5 or 6 or 7 or 8
10. Cancer Care Facilities/ or Oncology Service, Hospital/
11. exp Hospitals/
12. exp Hospital Administration/
13. inpatients/ or outpatients/ or exp Hospitalization/ or Home Care Services, Hospital-Based/
14. (hospital* or inpatient* or outpatient* or tertiary care or ward* or cancer care facilit* or cancer care unit* or cancer cent*).ti,ab.
15. 10 or 11 or 12 or 13 or 14
16. implementation science/ or (implement* or introduc*).ti,ab.
17. establish*.ti. or establishment.ti,ab.
18. "diffusion of innovation"/ or innovat*.ti.
19. Quality Improvement/ or quality improvement.ti,ab.
20. Health Plan Implementation/
21. "Delivery of Health Care, Integrated"/ or integrat*.ti,ab.
22. Program Development/ or development.ti,ab. or develop*.ti.
23. exp Evidence-Based Practice/
24. access*.ti. or (increase access* or early access).ti,ab.
25. (utilis* or utiliz*).ti.
26. *interdisciplinary communication/
27. Pilot Projects/ or Feasibility Studies/ or (pilot or feasibility).ti.
28. Perception/ or "Attitude of Health Personnel"/ or attitude*.ti.
29. "Referral and Consultation"/ or (refer* or consult* or early palliative).ti.
30. program evaluation/
31. (pathway* or barrier* or enable*).ti.
32. 16 or 17 or 18 or 19 or 20 or 21 or 22 or 23 or 24 or 25 or 26 or 27 or 28 or 29 or 30 or 31
33. 3 and 9 and 15 and 32
34. (child* or infant* or paediat* or pediat* or adolescen* or young or youth).ti.
35. 33 not 34
36. limit 35 to english language

Embase 1974 to 2021 March 10 (Ovid)

1. *palliative therapy/ or *cancer palliative therapy/ or *palliative nursing/ or *hospice/ or *hospice care/ or *hospice nursing/
2. (palliative or hospice* or "end of life care").ti,ab.
3. 1 or 2
4. exp *neoplasm/
5. exp *oncology/
6. *oncologist/ or *oncology nursing/
7. *oncology ward/ or *cancer center/
8. (cancer* or carcinoma* or leukaemia* or leukemia* or lymphoma* or malignan* or melanoma* or neoplasia* or neoplasm* or oncolog* or tumor* or tumour*).ti,ab.
9. 4 or 5 or 6 or 7 or 8
10. *oncology ward/ or *cancer center/
11. exp *hospital/
12. *hospital management/ or *hospital admission/ or *hospital planning/ or *hospital utilization/ or *hospitalization/
13. exp *hospital patient/
14. (hospital* or inpatient* or outpatient* or tertiary care or ward* or cancer care facilit* or cancer care unit* or cancer cent*).ti,ab.
15. 10 or 11 or 12 or 13 or 14
16. *implementation science/ or (implement* or introduc*).ti,ab.
17. establish*.ti. or establishment.ti,ab.
18. innovat*.ti.
19. *total quality management/ or quality improvement.ti,ab.
20. *integrated health care system/ or integrat*.ti,ab.
21. *program development/ or development.ti,ab. or develop*.ti.
22. exp *evidence based practice/
23. access*.ti. or (increase access* or early access).ti,ab.
24. (utilis* or utiliz*).ti.
25. *interdisciplinary communication/
26. *feasibility study/ or *pilot study/ or (pilot or feasibility).ti.
27. exp *health personnel attitude/ or attitude*.ti.
28. *patient referral/ or (refer* or consult* or early palliative).ti.
29. exp *program evaluation/
30. (pathway* or barrier* or enable*).ti.
31. 16 or 17 or 18 or 19 or 20 or 21 or 22 or 23 or 24 or 25 or 26 or 27 or 28 or 29 or 30
32. 3 and 9 and 15 and 31
33. (child* or infant* or paediat* or pediat* or adolescen* or young or youth).ti.
34. 32 not 33
35. limit 34 to english language

Ovid Emcare 1995 to 2021 Week 08

1. *palliative therapy/ or *cancer palliative therapy/ or *palliative nursing/ or *hospice/ or *hospice care/ or *hospice nursing/
2. (palliative or hospice* or "end of life care").ti,ab.
3. 1 or 2
4. exp *neoplasm/
5. exp *oncology/
6. *oncologist/ or *oncology nursing/
7. *oncology ward/ or *cancer center/

8. (cancer* or carcinoma* or leukaemia* or leukemia* or lymphoma* or malignan* or melanoma* or neoplasia* or neoplasm* or oncolog* or tumor* or tumour*).ti,ab.
9. 4 or 5 or 6 or 7 or 8
10. *oncology ward/ or *cancer center/
11. exp *hospital/
12. *hospital management/ or *hospital admission/ or *hospital planning/ or *hospital utilization/ or *hospitalization/
13. exp *hospital patient/
14. (hospital* or inpatient* or outpatient* or tertiary care or ward* or cancer care facilit* or cancer care unit* or cancer cent*).ti,ab.
15. 10 or 11 or 12 or 13 or 14
16. *implementation science/ or (implement* or introduc*).ti,ab.
17. establish*.ti. or establishment.ti,ab.
18. innovat*.ti.
19. *total quality management/ or quality improvement.ti,ab.
20. *integrated health care system/ or integrat*.ti,ab.
21. *program development/ or development.ti,ab. or develop*.ti.
22. exp *evidence based practice/
23. access*.ti. or (increase access* or early access).ti,ab.
24. (utilis* or utiliz*).ti.
25. *interdisciplinary communication/
26. *feasibility study/ or *pilot study/ or (pilot or feasibility).ti.
27. exp *health personnel attitude/ or attitude*.ti.
28. *patient referral/ or (refer* or consult* or early palliative).ti.
29. exp *program evaluation/
30. (pathway* or barrier* or enable*).ti.
31. 16 or 17 or 18 or 19 or 20 or 21 or 22 or 23 or 24 or 25 or 26 or 27 or 28 or 29 or 30
32. 3 and 9 and 15 and 31
33. (child* or infant* or paediat* or pediat* or adolescen* or young or youth).ti.
34. 32 not 33
35. limit 34 to english language

APA PsycInfo 1806 to March Week 1 2021 (Ovid)

1. palliative care/ or hospice/
2. (palliative or hospice* or "end of life care").ti,ab.
3. 1 or 2
4. exp Neoplasms/ or oncology/
5. (cancer* or carcinoma* or leukaemia* or leukemia* or lymphoma* or malignan* or melanoma* or neoplasia* or neoplasm* or oncolog* or tumor* or tumour*).ti,ab.
6. 4 or 5
7. hospitalization/ or hospitalized patients/
8. hospitals/ or hospital administration/
9. (hospital* or inpatient* or outpatient* or tertiary care or ward* or cancer care facilit* or cancer care unit* or cancer cent*).ti,ab.
10. 7 or 8 or 9
11. (implement* or introduc*).ti,ab.
12. establish*.ti. or establishment.ti,ab.
13. innovation/ or innovat*.ti.
14. "Quality of Care"/ or quality improvement.ti,ab.
15. integrated services/ or integrat*.ti,ab.

16. program development/ or development.ti,ab. or develop*.ti.
17. evidence based practice/
18. access*.ti. or (increase access* or early access).ti,ab.
19. (utilis* or utiliz*).ti.
20. (pilot or feasibility).ti.
21. health personnel attitudes/ or attitude*.ti.
22. professional referral/ or (refer* or consult* or early palliative).ti.
23. program evaluation/
24. (pathway* or barrier* or enable*).ti.
25. 11 or 12 or 13 or 14 or 15 or 16 or 17 or 18 or 19 or 20 or 21 or 22 or 23 or 24
26. 3 and 6 and 10 and 25
27. (child* or infant* or paediat* or pediat* or adolescen* or young or youth).ti.
28. 26 not 27
29. limit 28 to english language

CINAHL (EBSCOhost)

S1. (MH "Palliative Care") OR (MH "Hospice and Palliative Nursing") OR (MH "Hospice Care") OR (MH "Hospices")
S2. palliative OR hospice* OR "end of life care"
S3. S1 OR S2
S4. (MH "Neoplasms+")
S5. MH "Oncology+")
S6. (MH "Oncologists") OR (MH "Oncologic Nursing+") OR (MH "Radiation Oncology Nursing")
S7. (MH "Cancer Care Facilities") OR (MH "Oncology Care Units")
S8. cancer* OR carcinoma* OR leukaemia* OR leukemia* OR lymphoma* OR malignan* OR melanoma* OR neoplasia* OR neoplasm* OR oncolog* OR tumor* OR tumour*
S9. S4 OR S5 OR S6 OR S7 OR S8
S10. (MH "Cancer Care Facilities") OR (MH "Oncology Care Units")
S11. (MH "Hospitals+") OR (MH "Inpatients") OR (MH "Outpatients") OR (MH "Hospitalization")
S12. hospital* OR inpatient* OR outpatient* OR "tertiary care" OR ward* OR "cancer care facilit*" OR "cancer care unit*" OR "cancer cent*"
S13. S10 OR S11 OR S12
S14. (MH "Implementation Science") OR (MH "Program Implementation") OR (MH "Diffusion of Innovation")
S15. implement* OR introduc* OR TI innovat* OR TI establish* OR establishment
S16. (MH "Quality Improvement") OR "quality improvement"
S17. (MH "Health Care Delivery, Integrated") OR integrat*
S18. (MH "Program Development+") OR (MH "Program Evaluation") OR (MH "Program Planning") OR development OR TI develop*
S19. (MH "Professional Practice, Evidence-Based+")
S20. TI access* OR "increase access" OR "early access"
S21. TI utilis* OR TI utiliz*
S22. (MH "Pilot Studies") OR TI pilot OR TI feasibility
S23. (MH "Perception") OR (MH "Attitude of Health Personnel+") OR TI attitude*
S24. (MH "Attitude of Health Personnel+")
S25. (MH "Referral and Consultation") OR TI refer* OR TI consult* OR TI early palliative
S26. TI pathway* OR TI barrier* OR TI enable*

S27. S14 OR S15 OR S16 OR S17 OR S18 OR S19 OR S20 OR S21 OR S22 OR S23 OR S24 OR S25 OR S26
S28. S3 AND S9 AND S13 AND S27
S29. TI child* OR TI infant* OR TI paediat* OR TI pediat* OR TI adolescen* OR TI young OR TI youth
S30. S28 NOT S29 [limited to English language]

Cochrane Library (Wiley)

#1. [mh ^"Palliative Care"] OR [mh ^"Palliative Medicine"] OR [mh ^"Hospice and Palliative Care Nursing"] OR [mh ^Hospices] OR [mh ^"Hospice Care"]
#2. (palliative OR hospice* OR "end of life care"):ti,ab
#3. #1 OR #2
#4. [mh Neoplasms]
#5. [mh "Medical Oncology"]
#6. [mh Oncologists] OR [mh ^"Oncology Nursing"]
#7. [mh ^"Cancer Care Facilities"] OR [mh ^"Oncology Service, Hospital"]
#8. (cancer* OR carcinoma* OR leukaemia* OR leukemia* OR lymphoma* OR malignan* OR melanoma* OR neoplasia* OR neoplasm* OR oncolog* OR tumor* OR tumour*):ti,ab
#9. #4 OR #5 OR #6 OR #7 OR #8
#10. [mh ^"Cancer Care Facilities"] OR [mh ^"Oncology Service, Hospital"]
#11. [mh Hospitals]
#12. [mh "Hospital Administration"]
#13. [mh ^inpatients] OR [mh ^outpatients] OR [mh Hospitalization] OR [mh ^"Home Care Services, Hospital-Based"]
#14. (hospital* OR inpatient* OR outpatient* OR "tertiary care" OR ward* OR ("cancer care" NEAR/2 facilit*) OR ("cancer care" NEAR/2 unit*) OR ("cancer" NEAR/2 cent*)):ti,ab
#15. #10 OR #11 OR #12 OR #13 OR #14
#16. [mh ^"implementation science"] OR (implement* OR introduc*):ti,ab
#17. establish*:ti OR establishment:ti,ab
#18. [mh ^"diffusion of innovation"] OR innovat*:ti
#19. [mh ^"Quality Improvement"] OR "quality improvement":ti,ab
#20. [mh ^"Health Plan Implementation"]
#21. [mh ^"Delivery of Health Care, Integrated"] OR integrat*:ti,ab
#22. [mh ^"Program Development"] OR development:ti,ab OR develop*:ti
#23. [mh "Evidence-Based Practice"]
#24. access*:ti OR (("increase" NEAR/2 access*) OR "early access"):ti,ab
#25. (utilis* OR utiliz*):ti
#26. [mh ^"interdisciplinary communication"]
#27. [mh ^"Pilot Projects"] OR [mh ^"Feasibility Studies"] OR (pilot OR feasibility):ti
#28. [mh ^Perception] OR [mh ^"Attitude of Health Personnel"] OR attitude*:ti
#29. [mh ^"Referral and Consultation"] OR (refer* OR consult* OR "early palliative"):ti
#30. [mh ^"program evaluation"]
#31. (pathway* OR barrier* OR enable*):ti
#32. #16 OR #17 OR #18 OR #19 OR #20 OR #21 OR #22 OR #23 OR #24 OR #25 OR #26 OR #27 OR #28 OR #29 OR #30 OR #31
#33. #3 AND #9 AND #15 AND #32
#34. (child* OR infant* OR paediat* OR pediat* OR adolescen* OR young OR youth):ti
#35. #33 NOT #34
