# Supplementary material for: An evidence-base for the implementation of hospital-based palliative care programs in routine cancer practice: A systematic review
Source: Palliat Med. 2023 Jul 8;37(9):1326–44. doi: 10.1177/02692163231186177 (PMC10548767; doi:10.1177/02692163231186177)
Supplement: sj-pdf-2-pmj-10.1177_02692163231186177 – Supplemental material for An evidence-base for the implementation of hospital-based palliative care programs in routine cancer practice: A systematic review [file sj-pdf-2-pmj-10.1177_02692163231186177.pdf]

Supplementary File 3: Latest Search strategy for Ovid MEDLINE ALL 2021 to May 2023 (current)

#	Searches	Results
1	("palliative care"[Mesh:NoExp] OR palliative[tiab]) AND (neoplasms[Majr] OR cancer*[tiab]) AND hospital*[tiab] AND ((implement*[tiab] OR integrat*[tiab]) OR "implementation science"[Mesh:NoExp] {Including Related Terms}	0
2	limit 1 to (english language and full text and humans and yr="2021 -Current")	0
3	palliative care OR palliative AND neoplasms OR cancer* AND hospital* AND implement* OR integrat* OR "implementation science" {Including Related Terms}	10010
4	limit 3 to (english language and full text and humans and yr="2021 -Current")	181

After title and abstract search, 8 papers were screened full-text, and 1 paper has been selected to be included in the results table from the year 2022
